# Gollin's (1965) levels-by-levels approach: the importance of manipulating the task dimension when assessing age-related changes and individual differences in decision making

**DOI:** 10.3389/fpsyg.2015.00541

**Published:** 2015-04-30

**Authors:** Kana Imuta, Josh Hewitt, Damian Scarf

**Affiliations:** ^1^Department of Psychology, University of QueenslandBrisbane, QLD, Australia; ^2^Department of Psychology, University of OtagoDunedin, New Zealand

**Keywords:** cognitive development, child development, decision making, delay of gratification, contextual variables, levels by levels approach, behavioral economics

The growing interest in *individual* differences in decision making has been accompanied by a growing interest in *developmental* differences. A developmental perspective has the power to elucidate the role that brain development and experience play in decision making. However, it also has the potential to mislead. In contrast to adult participants, who one assumes are similar in their understanding of an experimental paradigm, children of different ages may view a paradigm through very different lenses. Of course, the point of a developmental perspective is to investigate how decision making changes across development, but the findings are only valid in so far as each age group's understanding of the experimental paradigm is equivalent.

Gollin's (1965) levels-by-levels approach addresses this issue by highlighting the value of manipulating the “organismic dimension” (level 1) and “task dimension” (level 2) concurrently. While it is commonplace for developmental psychologists to manipulate the organismic dimension (i.e., the age of participants), the task dimension is often overlooked. This manipulation comes in one of two forms: Gollin's (1965) approach of systematically increasing task complexity and Bitterman's (1964) approach of identifying and controlling for “contextual variables” (i.e., factors other than the one of interest that adversely impact a subject's performance). Comparative psychology perhaps holds the clearest examples of these two approaches (Macphail, 1985). Gollin's (1965) systematic approach is exemplified by Lashley's (1929) investigation of the impact of brain lesions on maze learning in rats. Lashley (1929) systematically varied both the size of the lesion (i.e., the organismic dimension) and the complexity of the maze (i.e., the task dimension). The lesions had little impact on simple mazes, suggesting that basic learning was not impaired, but as the complexity of the mazes increased, the lesions had greater and greater impact and this impact was mediated by the size of the lesion.

The most marked example of Bitterman's (1964) approach derives from Harlow's (1949) learning set paradigm. The paradigm was designed to assess an animal's ability to “learn how to learn” and was initially thought to provide a way of ranking species by intelligence (e.g., Hodos, 1970; Jerison, 1973; Warren, 1974). In short, subjects are presented with a series of problems in which two stimuli are presented; selecting one stimulus results in a reward while selecting the other stimulus results in a punishment. Subjects are said to have developed a *learning set* once they start to select the rewarded stimulus on the second trial of each new problem (i.e., Win Stay: If rewarded on Trial 1, respond to the same stimulus on Trial 2; Lose Shift: If punished on Trial 1, respond to the other stimulus on Trial 2). Initial studies employing the learning set paradigm found marked species differences. Specifically, most primate species rapidly acquired a learning set, while rats continued to perform at chance after several thousand trials (Hodos, 1970). Slotnick and Katz (1974), however, demonstrated that rats trained with olfactory, rather than visual stimuli, acquired a learning set at a similar speed to chimpanzees. For rats, therefore, the visual modality of the stimuli used in the initial experiments was a contextual variable.

## Delay of gratification

We recently applied the levels-by-levels approach to the delay-of-gratification choice paradigm (Imuta et al., 2014). Mischel (1958) developed the choice paradigm prior to developing the well-known maintenance paradigm, colloquially referred to as the *Marshmallow Test* (see Mischel, 2014, for an overview). Similar to the maintenance paradigm, in the choice paradigm, participants are faced with a small reward that they can receive immediately (i.e., “Now”) or a larger reward that they can receive after a delay (i.e., “Later”). In contrast to the maintenance paradigm, where children must *maintain* their decision to wait for the larger reward, in the choice paradigm, children commit to either waiting or not at the start of each trial. The dependent variable is the proportion of trials on which children choose to wait. The general finding is that 3-year-olds fail to delay gratification on the choice paradigm, while 4-year-olds succeed (Thompson et al., 1997; Hongwanishkul et al., 2005; Prencipe and Zelazo, 2005; Lemmon and Moore, 2007).

**Figure 1:**
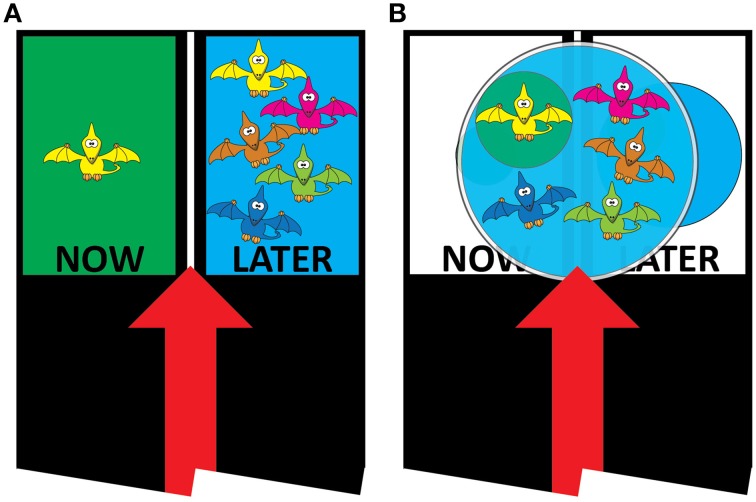
**The presentation of the sticker rewards in the (A) standard and (B) modified tasks**.

Building on the extensive literature varying the task dimension of the maintenance paradigm (e.g., Mischel and Ebbesen, 1970; Anderson, 1978; Karniol et al., 2011), Imuta et al. (2014) manipulated the way in which the immediate and delayed rewards were presented. Typically the immediate and delayed rewards are presented in two groups that are physically separated in space (e.g., Prencipe and Zelazo, 2005; Lemmon and Moore, 2007; Garon et al., 2011; see Figure 1A). When presented in this manner, should children choose the “Now” option, they can maintain their attention on the “Now” option (i.e., one sticker) and largely ignore the removal the “Later” option (i.e., five stickers). To circumvent this, Imuta et al. (2014) presented the stickers in a single group (see Figure 1B). By presenting the task in this manner, children can no longer ignore the quantity costs (i.e., four stickers) of selecting the “Now” option. Imuta et al. (2014) demonstrated that 4-year-olds chose the “Later” option at a level significantly above chance (i.e., 50%) on both the standard (75%) and modified (82%) paradigms. The performance of 3-year-olds, however, differed significantly between the two paradigms, relatively infrequently choosing the “Later” option on the standard paradigm (37%) but selecting it in the majority (76%) of trials on the modified paradigm. By manipulating the age of participants and the task (i.e., presentation format), we were able to demonstrate that the presentation format was a contextual variable for the younger, but not older, children.

## Decision making under risk

The development of decision making under risk is a rapidly growing area of research wherein the levels-by-levels approach has, and will continue to be, extremely valuable. For example, the Iowa Gambling Task (IGT) was initially developed to assess patients with prefrontal lesions (Bechara et al., 1994), but is now being used to study the development of affective decision making (e.g., Crone and van der Molen, 2004; Hooper et al., 2004; Cauffman et al., 2010; Van Duijvenvoorde et al., 2012). In the standard IGT, participants are presented with four decks of cards, each differing in their win:loss reward ratio. Participants are asked to draw from the decks with the aim of maximizing their reward. Two of the decks are “disadvantageous,” in that they give high-value rewards but also high losses, leading to an overall loss in the long term. The other two decks are “advantageous” decks, which yield lower rewards but also lower losses, resulting in an overall gain if participants consistently select cards from these decks.

The majority of developmental studies that have used the IGT have focused on simply charting changes in performance across age, generally noting that children below the age of 12 fail to zero in on the advantageous decks (Crone and van der Molen, 2004; Kerr and Zelazo, 2004; Overman et al., 2004; Garon and Moore, 2007). Only recently have researchers begun to investigate whether this age-related change is the result of developmental differences in decision making, or attributable to some contextual variable. For example, to investigate whether limitations in working memory, rather than decision making, can account for the developmental difference in IGT performance, Van Duijvenvoorde et al. (2012) compared participants' performance on an *informed IGT*, where the gains and losses associated with each gambling machine (i.e., equivalent of a deck) were explicitly presented on a computer screen, to that on a standard *non-informed IGT*, where participants had to keep track of the gains, losses, and probabilities associated with each machine. Consistent with previous research, Van Duijvenvoorde et al. (2012) found that children under the age of 12 failed to make advantageous decisions in the non-informed IGT. In contrast, children as young as 7 years of age made advantageous decisions on the informed IGT. By taking the levels-by-levels approach of manipulating both the age of participants and the task, Van Duijvenvoorde et al.'s (2012) study provides evidence that children as young as 7 years are capable of engaging in advantageous affective decision making, but also sheds light on the specific cognitive limitation (i.e., memory) that younger children face in this process. Future studies may look to combine Van Duijvenvoorde et al.'s (2012) approach with the play/pass version of the IGT, initially developed to investigate individual differences in response to gains and losses (Peters and Slovic, 2000; Cauffman et al., 2010), to provide a more nuanced account of age-related changes in decision making on the IGT.

Investigating the task dimension becomes even more important as studies of decision making test younger and younger children. Several groups have developed simplified decision making tasks with the aim of minimizing task difficulty for younger participants (Paulsen et al., 2011, 2012; Weller et al., 2011). For example, in the first study to assess economic decision making in pre-school children, Steelandt et al. (2013) assessed the rationality of choices and judgment errors made by 3- to 9-year-old children. Children were given an initial offering of a medium-sized piece of cookie and then choose either to keep that offer or risk it, by exchanging it for one of six cups that had more, less, or the same amount of cookie, chosen at random. The amount of cookie in each cup was manipulated such that on some trials, all six cups held more cookies (i.e., a guaranteed gain), and on other trials, the majority of the six cups held less.

Steelandt et al. (2013) found that 3- and 4-year-olds performed poorly while 5- and 6-year-olds were able to correctly distinguish profitable situations from non-profitable situations. The performance of the 3- to 4-year-olds may be due to economic decision-making not being present at this age, or the complexity of the paradigm. Indeed, the gamble option required children to keep track of the reward across six separate cups, maintain that information in memory, and use that information to estimate the probability of winning or losing. A levels-by-levels approach, in which the number of cups children must keep track of is manipulated, along with children's age, would allow one to disentangle these two possibilities. Similar to Lashley's (1929) rats, it may be that developmental differences in decision making under risk are closely tied to task complexity, such that developmental differences only emerge when the task reaches a certain level of complexity.

## Conclusion

As illustrated in the examples provided above, the key to gaining a developmental perspective of decision making hinges on paying careful attention to the interaction between the participant dimension and the task dimension. This has particular relevance for the study of individual differences, given it has the potential to inform whether the individual differences observed are a result of individual differences in decision making or individual differences with respect to task understanding. While the use of a single standardized task that can be used across ages and individuals is appealing, as the studies outlined above suggest, by putting all your eggs in one basket you may miss the nuances in the data that Gollin's (1965) levels-by-levels approach can provide.

### Conflict of interest statement

The authors declare that the research was conducted in the absence of any commercial or financial relationships that could be construed as a potential conflict of interest.

## References

[B1] Anderson W. H. (1978). A comparison of self-distraction with self-verbalization under moralistic versus instrumental rationales in a delay-of-gratification paradigm. Cognit. Ther. Res. 2, 299–303 10.1007/BF01185793

[B2] Bechara A. Damasio A. R. Damasio H. Anderson S. W. (1994). Insensitivity to future consequences following damage to human prefrontal cortext. Cognition 50, 7–15. 10.1016/0010-0277(94)90018-3 8039375

[B3] Bitterman M. E. (1964). The evolution of intelligence. Sci. Am. 212, 92–100. 10.1038/scientificamerican0165-92 14252463

[B4] Cauffman E. Shulman E. P. Steinberg L. Claus E. Banich M. T. Graham S.. (2010). Age differences in affective decision making as indexed by performance on the Iowa Gambling Task. Dev. Psychol. 46, 193–207. 10.1037/a0016128 20053017

[B5] Crone E. A. van der Molen M. W. (2004). Developmental changes in real life decision making: performance on a gambling task previously shown to depend on the ventromedial prefrontal cortex. Dev. Neuropsychol. 25, 251–279. 10.1207/s15326942dn2503_2 15147999

[B6] Garon N. Moore C. (2007). Awareness and symbol use improves future-oriented decision making in pre-schoolers. Dev. Neuropsychol. 31, 39–59. 10.1207/s15326942dn3101_3 17305437

[B7] Garon N. Johnson B. Steeves A. (2011). Sharing with others and delaying for the future in preschoolers. Cogn. Dev. 26, 383–396 10.1016/j.cogdev.2011.09.007

[B8] Gollin E. S. (1965). A developmental approach to learning and cognition. Adv. Child Dev. Behav. 2, 159–186 10.1016/S0065-2407(08)60482-6

[B9] Harlow H. F. (1949). The formation of learning sets. Psychol. Rev. 56, 51–65 10.1037/h0062474 18124807

[B10] Hodos W. (1970). Evolutionary interpretation of neural and behavioral studies in living vertebrates, in The Neurosciences: Second Study Program, ed Schmidt F. O. (New York, NY: Rockefeller University Press), 26–39.

[B11] Hongwanishkul D. Happaney K. R. Lee W. S. Zelazo P. D. (2005). Assessment of hot and cool executive function in young children: age-related changes and individual differences. Dev. Neuropsychol. 28, 617–644. 10.1207/s15326942dn2802_4 16144430

[B12] Hooper C. J. Luciana M. Conklin H. M. Yarger R. S. (2004). Adolescents' performance on the Iowa Gambling Task: implications for the development of decision making and ventromedial prefrontal cortex. Dev. Psychol. 40, 1148–1158. 10.1037/0012-1649.40.6.1148 15535763

[B13] Imuta K. Hayne H. Scarf D. (2014). I want it all and I want it now: delay of gratification in preschool children. Dev. Psychobiol. 56, 1541–1552. 10.1002/dev.21249 25139433

[B14] Jerison H. J. (1973). Evolution of the Brain and Intelligence. New York, NY: Academic Press.

[B15] Karniol R. Galili L. Shtilerman D. Naim R. Stern K. Manjoch H.. (2011). Why Superman can wait: cognitive self-transformation in the delay of gratification paradigm. J. Clin. Child Adolesc. Psychol. 40, 307–317. 10.1080/15374416.2011.546040 21391026

[B16] Kerr A. Zelazo P. D. (2004). Development of “hot” executive function: the children's gambling task. Brain Cogn. 55, 148–157. 10.1016/S0278-2626(03)00275-6 15134849

[B17] Lashley K. S. (1929). Brain Mechanisms and Intelligence: A Quantitative Study of Injuries to the Brain. Chicago, IL: Chicago University Press 10.1037/10017-000

[B18] Lemmon K. Moore C. (2007). The development of prudence in the face of varying future rewards. Dev. Sci. 10, 502–511. 10.1111/j.1467-7687.2007.00603.x 17552939

[B19] Macphail E. (1985). Vertebrate intelligence: the null hypothesis. Philos. Trans. R. Soc. B Biol. Sci. 308, 37–51 10.1098/rstb.1985.0008

[B20] Mischel W. (1958). Preference for delayed reinforcement: an experimental study of a cultural observations. J. Abnorm. Soc. Psychol. 56, 57–61. 10.1037/h0041895 13501972

[B21] Mischel W. (2014). The Marshmallow Test: Mastering Self-control. New York, NY: Hachette Book Group, Inc.

[B22] Mischel W. Ebbesen E. B. (1970). Attention in delay of gratification. J. Pers. Soc. Psychol. 16, 329–337 10.1037/h0029815 5010404

[B23] Overman W. H. Frassrand K. Ansel S. Trawalter S. Bies B. Redmond A. (2004). Performance on the Iowa card task by adolescents and adults. Neuropsychologia 42, 1838–1851. 10.1016/j.neuropsychologia.2004.03.014 15351632

[B24] Paulsen D. J. Carter R. M. Platt M. L. Huettel S. A. Brannon E. M. (2011). Neurocognitive development of risk aversion from early childhood to adulthood. Front. Hum. Neurosci. 5:178. 10.3389/fnhum.2011.00178 22291627 PMC3250075

[B25] Paulsen D. J. Platt M. L. Huettel S. A. Brannon E. M. (2012). From risk-seeking to risk-averse: the development of economic risk preference from childhood to adulthood. Front. Psychol. 3:313. 10.3389/fpsyg.2012.00313 22973247 PMC3436335

[B26] Peters E. Slovic P. (2000). The springs of action: affective and analytical information processing in choice. Pers. Soc. Psychol. Bull. 26, 1465–1475 10.1177/01461672002612002

[B27] Prencipe A. Zelazo P. D. (2005). Development of affective decision making for self and other: evidence for the integration of first- and third-person perspectives. Psychol. Sci. 16, 501–505. 10.1111/j.0956-7976.2005.01564.x 16008779

[B28] Slotnick B. M. Katz H. M. (1974). Olfactory learning-set formation in rats. Science 30, 796–798 10.1126/science.185.4153.796 4843380

[B29] Steelandt S. Broihanne M.-H. Romain A. Thierry B. Dufour V. (2013). Decision-making under risk of loss in children. PLoS ONE 8:e52316. 10.1371/journal.pone.0052316 23349682 PMC3548654

[B30] Thompson C. Barresi J. Moore C. (1997). The development of future-oriented prudence and altruism in preschoolers. Early Child Res. Q. 12, 199–212 10.1016/S0885-2014(97)90013-712294306

[B31] Van Duijvenvoorde A. C. K. Jansen B. R. J. Bredman J. C. Huizenga H. M. (2012). Age-related changes in decision making: comparing informed and noninformed situations. Dev. Psychol. 48, 192–203. 10.1037/a0025601 21967563

[B32] Warren J. M. (1974). Possibly unique characteristics of learning by primates. J. Hum. Evol. 3, 445–454. 10.1016/0047-2484(74)90004-924172288

[B33] Weller J. A. Levin I. P. Denburg N. L. (2011). Trajectory of risky decision making for potential gains and losses from ages 5 to 85. J. Behav. Decis. Mak. 24, 331–344 10.1002/bdm.690

